# Inhibition of the SEC61 translocon by mycolactone induces a protective autophagic response controlled by EIF2S1-dependent translation that does not require ULK1 activity

**DOI:** 10.1080/15548627.2021.1961067

**Published:** 2021-08-23

**Authors:** Belinda S Hall, Scott J Dos Santos, Louise Tzung-Harn Hsieh, Maria Manifava, Marie-Thérèse Ruf, Gerd Pluschke, Nicholas Ktistakis, Rachel E Simmonds

**Affiliations:** a Department of Microbial Sciences, School of Biosciences and Medicine, University of Surrey, Guildford, UK; b Signaling Programme, Babraham Institute, Cambridge, UK; c Molecular Immunology, Swiss Tropical and Public Health Institute, Basel, Switzerland; d Medical Parasitology and Infection Biology Department, University of Basel, Basel, Switzerland

**Keywords:** Buruli ulcer, eif2s1/eIF2α, integrated stress response, mislocalized proteins, mycolactone, RB1CC1/FIP200, SQSTM1/p62, translocation inhibitor, ulk1

## Abstract

The *Mycobacterium ulcerans* exotoxin, mycolactone, is responsible for the immunosuppression and tissue necrosis that characterizes Buruli ulcer. Mycolactone inhibits SEC61-dependent co-translational translocation of proteins into the endoplasmic reticulum and the resultant cytosolic translation triggers degradation of mislocalized proteins by the ubiquitin-proteasome system. Inhibition of SEC61 by mycolactone also activates multiple EIF2S1/eIF2α kinases in the integrated stress response (ISR). Here we show mycolactone increased canonical markers of selective macroautophagy/autophagy LC3B-II, ubiquitin and SQSTM1/p62 in diverse disease-relevant primary cells and cell lines. Increased formation of puncta positive for the early autophagy markers WIPI2, RB1CC1/FIP200 and ATG16L1 indicates increased initiation of autophagy. The mycolactone response was SEC61A1-dependent and involved a pathway that required RB1CC1 but not ULK. Deletion of *Sqstm1* reduced cell survival in the presence of mycolactone, suggesting this response protects against the increased cytosolic protein burden caused by the toxin. However, reconstitution of baseline SQSTM1 expression in cells lacking all autophagy receptor proteins could not rescue viability. Translational regulation by EIF2S1 in the ISR plays a key role in the autophagic response to mycolactone. Mycolactone-dependent induction of SQSTM1 was reduced in *eif2ak3^−/-^/perk*^−/-^ cells while the p-EIF2S1 antagonist ISRIB reversed the upregulation of SQSTM1 and reduced RB1CC1, WIPI2 and LC3B puncta formation. Increased SQSTM1 staining could be seen in Buruli ulcer patient skin biopsy samples, reinforcing genetic data that suggests autophagy is relevant to disease pathology. Since selective autophagy and the ISR are both implicated in neurodegeneration, cancer and inflammation, the pathway uncovered here may have a broad relevance to human disease.

**Abbreviations:** ATF4: activating transcription factor 4; ATG: autophagy related; BAF: bafilomycin A_1_; ATG16L1: autophagy related 16 like 1; BU: Buruli ulcer; CQ: chloroquine; EIF2AK3: eukaryotic translation initiation factor 2 alpha kinase 3; CALCOCO2: calcium binding and coiled-coil domain 2; DMSO: dimethyl sulfoxide; EIF2S1: eukaryotic translation initiation factor 2 subunit alpha; ER: endoplasmic reticulum; GFP: green fluorescent protein; HDMEC: human dermal microvascular endothelial cells; HFFF: human fetal foreskin fibroblasts; ISR: integrated stress response; ISRIB: integrated stress response inhibitor; MAP1LC3B/LC3B: microtubule associated protein 1 light chain 3 beta; MEF: mouse embryonic fibroblast; Myco: mycolactone; NBR1: NBR1 autophagy cargo receptor; NFE2L2: nuclear factor, erythroid 2 like 2; OPTN: optineurin; PFA: paraformaldehyde; PtdIns3P: phosphatidylinositol-3-phosphate; RB1CC1: RB1-inducible coiled coil 1; SQSTM1: sequestosome 1; TAX1BP1: Tax1 binding protein 1; ULK: unc-51 like autophagy activating kinase; UPS: ubiquitin-proteasome system; WIPI: WD repeat domain, phosphoinositide interacting; WT: wild type.

## Introduction

The polyketide exotoxin, mycolactone, produced by *Mycobacterium ulcerans*, is the sole driver of the pathology associated with the neglected tropical disease, Buruli ulcer [1,2] (BU). The disease is a chronic skin infection characterized by progressive necrosis that can lead to permanent disfigurement and disability. Mycolactone alone can induce necrotic lesions if injected into the skin. As well as being cytotoxic, mycolactone is a potent immunosuppressant and is responsible for the downregulation of immune responses seen in BU, acting via direct immune cell destruction and, at sub-cytotoxic doses, an inhibition of antigen presentation, co-stimulation and cytokine secretion [3–5]. Consequently, even extensive lesions display very little macroscopic signs of inflammation despite a heavy bacterial load. Mycolactone also causes endothelial cell dysfunction, disrupting the control of coagulation pathways [6].

The primary target of mycolactone is now known to be the SEC61 translocon [7,8], a heterotrimeric channel enabling co-translational translocation of proteins into the endoplasmic reticulum (ER), necessary for correct localization of transmembrane proteins and transit of secretory proteins through the cell. Even at nanomolar concentrations, mycolactone inhibits translocation of secretory proteins, type II transmembrane proteins and almost all type I transmembrane proteins into the ER via direct interaction with the core component SEC61A1/Sec61α (SEC61 translocon subunit alpha 1) [7,9,10]. These observations provide a robust explanation for the previously reported consequences of its activity, including rounding-up and detachment of cells from culture surfaces *in vitro*, downregulation of immune responses, and eventually, cell death [11–15]. We and others recently demonstrated the ability of mycolactone to activate the integrated stress response (ISR) [16,17]. Phosphorylation of the translation initiation factor EIF2S1/eIF2α (eukaryotic translation initiation factor 2 subunit alpha) causes a global inhibition of translation but, for some transcripts such as the transcription factor ATF4 (activating transcription factor 4), translation is activated by a well-characterized mechanism involving upstream ORFs [18]. Mycolactone activates multiple EIF2S1 kinases including EIF2AK3/PERK (eukaryotic translation initiation factor 2 alpha kinase 3), leading to increased ATF4 expression and induction of its downstream target DDIT3/CHOP (DNA damage inducible transcript 3), an apoptosis regulator [16,17]. In many cell types, cell death does not occur until 4–5 d after initial exposure [6,16]. The induction of ATF4 and subsequent cell death is dependent on inhibition of co-translational translocation and is prevented by expression of certain SEC61A1 mutants that render cells resistant to mycolactone [11,16].

When the SEC61 complex is inhibited (hereafter referred to as SEC61 inhibition), proteins that depend on co-translational translocation are instead synthesized in the cytosol, where they are targeted to the ubiquitin-proteasome system (UPS) [7,19]. Mislocalized ER proteins are recognized by cytosolic proteins such as the chaperone BAG6 (BAG chaperone 6), leading to their ubiquitination [20] and degradation by the 26S proteasome [21], but macroautophagy/autophagy (hereafter referred to as autophagy) can also play a role in removal of ubiquitinated proteins. While once thought to be a bulk-degradation process, autophagy is now known to exhibit selectivity, governed by specific autophagic receptor proteins, such as SQSTM1 (sequestosome 1) [22].

Autophagic membranes (phagophores) originate from a phosphatidylinositol-3-phosphate (PtdIns3P)-rich subdomain of the ER: the omegasome. To date it has generally been considered that in canonical autophagy, production of these domains is regulated by the ULK (unc-51 like autophagy activating kinase) complex, consisting of ULK1 or ULK2, RB1CC1/FIP200 (RB1-inducible coiled coil 1), ATG101 and ATG13 [23,24]. ULK activity is regulated by the nutritional state of cells via the activity of MTOR (mechanistic target of rapamycin kinase). Phosphorylation by the MTOR complex 1 (MTORC1) at Ser757 in mice (Ser758 in humans) maintains ULK1 in an inactive state under normal conditions but during nutrient deprivation, reduced MTORC1 activity results in ULK1 activation to promote autophagy, recover amino acids via protein digestion and restore homeostasis [23]. ULK phosphorylates and activates the BECN1 (beclin 1)-PIK3C3/VPS34 (phosphatidylinositol 3-kinase catalytic subunit type 3) complex, leading to increased PtdIns3P on the omegasome membrane [23,25]. Here, WIPI (WD repeat domain, phosphoinositide interacting) proteins such as WIPI2 bind to PtdIns3P at the surface of the expanding phagophore. WIPI2 interacts with ATG16L1 (autophagy related 16 like 1), one of the proteins in the conjugating complex that lipidates Atg8-family proteins, of which MAP1LC3B/LC3B (microtubule associated protein 1 light chain 3 beta) is the most well-characterized [26], thus leading to their recruitment at the site of phosphatidylinositol phosphorylation. Phosphatidylethanolamine-conjugated LC3B (LC3B-II) serves as a docking site for autophagy receptors. In selective autophagy, the interaction between SQSTM1 (or other receptors such as CALCOCO2 [calcium binding and coiled-coil domain 2], OPTN [optineurin], NBR1 [NBR1 autophagy cargo receptor] and TAX1BP1 [Tax1 binding protein 1]) and LC3B is central to the degradation of protein aggregates [27–30]. Ubiquitinated aggregates bind to SQSTM1 which in turn binds to LC3B [31]. Closure of the autophagosome membrane encapsulates the receptor-bound cargo which is then degraded on fusion with the lysosome.

In our previous investigation [16] one of the transcripts that showed an increase in translation in response to 5 h mycolactone exposure was that encoding SQSTM1. Another published proteomic dataset also reported increased SQSTM1 abundance after 24 h [13]. Taken together, this suggested that, in addition to activation of the UPS, accumulation of mislocalized proteins in the cytosol due to mycolactone may also trigger an increase in selective autophagy [9,12]. Moreover, we previously showed that exposure of cells to mycolactone caused increased LC3B lipidation and puncta formation [16]. However, others have reported that mycolactone inhibits autophagy [19]. To resolve this conflict and establish the role of autophagy in mycolactone toxicity we have carried out a detailed investigation of the autophagic flux changes in mycolactone-exposed cells and the cellular mechanisms driving them. Here, we show that in addition to an increase in LC3B puncta formation and protein ubiquitination [16,19], mycolactone triggers an increase in initiation of autophagy. We highlight the role of the selective autophagy marker SQSTM1 in the response and show that mycolactone-induced autophagy initiation does not require the ULK1 and ULK2 proteins but otherwise depends on the canonical autophagy machinery (including the RB1CC1 protein and the omegasome intermediates) and provide evidence for translational regulation of these pathways through EIF2S1 phosphorylation.

## Results

### Mycolactone induces an increase in autophagic flux

**Figure 1.**
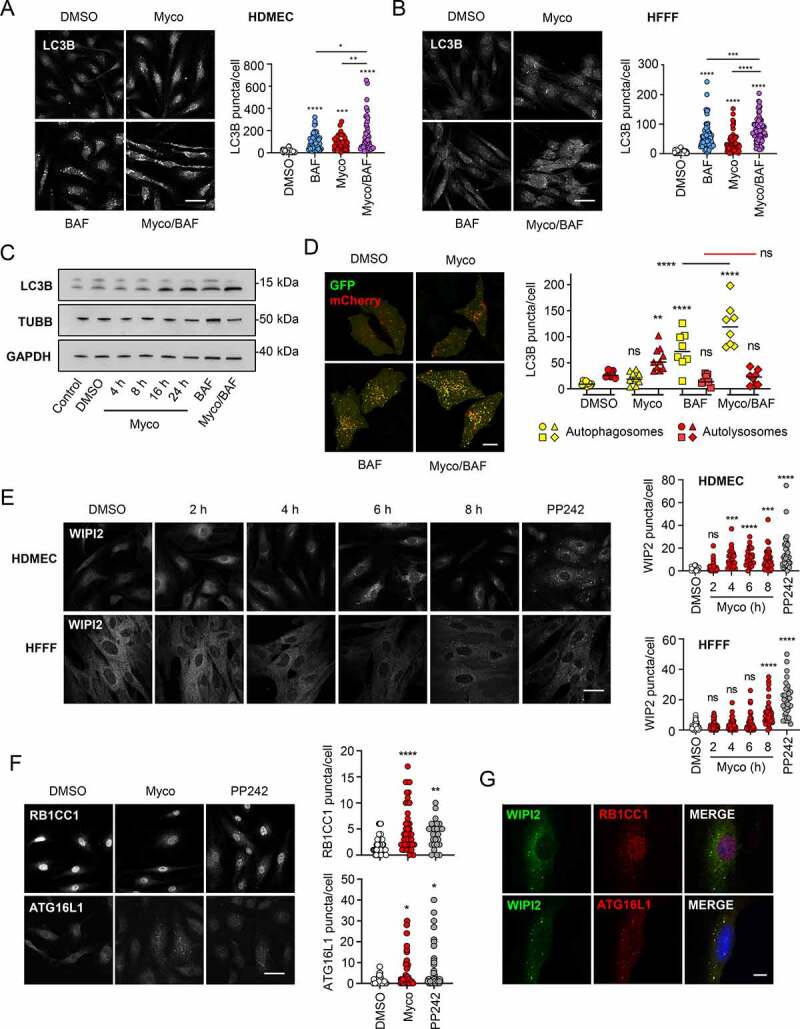
Mycolactone induces an increase in autophagic flux in primary dermal microvascular endothelial cells and fibroblasts. Cells were exposed to mycolactone (Myco) at the minimal inhibitory concentration alongside a solvent control (0.05% DMSO, equivalent dilution to the mycolactone) for the longest timepoint tested. where included, bafilomycin A_1_ (BAF) was used for 4 h at cell-specific saturating concentrations, and at the longest timepoint tested when in combination with mycolactone. PP242 was used at 1 μM for 1 h. All immunofluorescence images were obtained using a Nikon A1 confocal laser scanning microscope. For all panels, unless otherwise indicated, statistical comparisons are to the DMSO-treated cells of that particular cell type; ns = not significant; * = *p*< 0.05; ** = *p*< 0.01; *** = p < 0.001; **** = p < 0.0001. (A) HDMEC exposed to 10 ng/ml mycolactone with and without 100 nM BAF for 8 h were fixed with methanol for LC3B staining (scale bar: 50 µm). values represent the number of puncta detected per cell for at least 36–49 cells. Data are representative of 2 independent experiments (scale bar: 50 µm). (B) HFFF exposed to 31.25 ng/ml mycolactone with and without 200 nM BAF for 8 h were fixed with methanol for LC3B staining (scale bar: 50 µm). Values represent the number of puncta detected per cell for at least 63–90 cells. Data are representative of 2 independent experiments. (scale bar: 50 µm) experiments. (C) HFFF cells exposed to 31.25 ng/ml mycolactone and/or 200 nM BAF were lysed directly in gel sample buffer prior to immunoblotting. The migration relative to known molecular mass markers is shown. Data are representative of 2 independent experiments. (D) Expression of tandem-tagged LC3B in HeLa cells. Cells transiently transfected with pDEST-mCherry-eGFP-LC3B were exposed to mycolactone for 4 h before the addition of 100 nM (BAF) and incubation for a further 4 h before fixation. Values represent the number of puncta detected per cell for at least 8–10 transfected cells. Data are representative of 4 independent experiments (scale bar: 10 μm). (E) HDMEC and HFFF were exposed to mycolactone for various times or PP242, then fixed for WIPI2 staining (scale bar: 50 µm). Values represent the puncta detected in each cell for 25–50 cells. Data are representative of 2 independent experiments. (F) HDMEC were exposed to 10 ng/ml mycolactone for 8 h, or PP242 then fixed for RB1CC1 or ATG16L1 staining (scale bar: 50 µm). Values represent the puncta detected in each cell for 25–50 cells. Data are representative of 2 independent experiments. (G) HDMEC were exposed to 10 ng/ml mycolactone for 8 h then fixed for co-staining with WIPI (green) and RB1CC1 or ATG16L1 (red) (scale bar: 10 μm).

We have previously shown an increase in LC3B lipidation in HeLa cells in response to mycolactone [16]. To determine whether this response also occurs in disease-relevant primary cells from skin, we examined LC3B puncta formation in human dermal microvascular endothelial cells (HDMEC), which are highly sensitive to the effects of mycolactone and may play a role in disease pathology [6], and in primary human fetal foreskin fibroblasts (HFFF) by immunofluorescence microscopy. In both cell types, exposure to mycolactone for 8 h induced a significant increase in cytosolic LC3B puncta compared to the DMSO vehicle (Figure 1A and 1B). Similar results were obtained with both murine embryonic fibroblasts (MEF) and HeLa cell lines, although the kinetics were slower in the latter, requiring 16 h to reach a significant increase (**Fig. S1A and S1B**). As expected, LC3B puncta were increased by incubation with saturating levels of either bafilomycin A_1_ (or chloroquine; **Fig. S1B**) and co-incubation with mycolactone led to a further increase(Figure 1A,1B **and S1B**). A time-dependent increase in lipidated LC3B-II was detected in HFFF cells exposed to mycolactone and this was further increased in the presence of bafilomycin A_1_ (Figure 1C, quantified in **Fig. S1C**).

To determine whether this observation was due to a *bona fide* activation of autophagy or a decrease in the turnover of autophagosomes owing to inhibition of the terminal degradative step in the pathway, we utilized a previously described [31] mCherry-GFP-LC3B tandem tag to follow the fusion of autophagosomes with the lysosome. HeLa cells transfected with the tandem tag construct showed a basal level of autophagy, with detection of both red and yellow puncta in cells incubated with DMSO (Figure 1D **and S1D**). As expected, no puncta formation was detected in cells expressing a control mCherry-GFP (**Fig. S1E**). Exposure of cells transfected with a plasmid encoding the LC3B-fusion protein to mycolactone had little effect on the number of autophagosomes (yellow puncta), but significantly increased the number of LC3B-positive autolysosomes (red puncta), suggesting that mycolactone does not block fusion of autophagosomes with lysosomes or their acidification (Figure 1D). Addition of a saturating concentration of bafilomycin A_1_ (**Fig. S1F**) reduced the number of acidified red structures while also causing an accumulation of neutral, yellow puncta, as expected. Co-incubation with mycolactone and bafilomycin A_1_ slightly increased levels of acidified vesicles while significantly increasing the number of neutral, yellow autophagic vesicles compared to bafilomycin A_1_ alone (Figure 1D). Taken together this data suggests that mycolactone does indeed induce an increase in autophagic flux.

As an alternative approach to assessing changes in autophagic flux we also assessed mycolactone’s ability to promote phagophore formation by following appearance of WIPI2 puncta by immunofluorescence staining [32]. In both HDMEC and HFFF, WIPI2 puncta increased with time of exposure to mycolactone (Figure 1E). The response is slower than that induced by the MTOR inhibitor PP242 [33], but a significant increase in puncta could be detected at 4 h in HDMEC and 8 h in HFFF cells. Similar changes were observed in MEF and HeLa cell lines (**Fig. S1G and S1H**). The production of early autophagosomes was further assessed by staining HDMEC cells for RB1CC1 and ATG16L1. Both markers showed an increase in puncta comparable to the positive control PP242 8 h after mycolactone addition (Figure 1F). Co-staining revealed close association of both RB1CC1 and ATG16L1 with WIPI2 in HDMEC and MEFs, suggesting they were present on the same structures (Figure 1G **and S1H**). RB1CC1 and ATG16L1 puncta were also detected in HFFF cells but the effect was less marked (**Fig. S1J**). Since all three markers are only transiently associated with autophagosomes and (unlike LC3B) are not destroyed by fusion with the lysosome, the most likely explanation for the increase in puncta detection is an increase in autophagic flux. However, a “dual effect” of an increase in autophagic flux together with inhibition of autophagosome maturation cannot be completely ruled out at this stage.

### Mycolactone-induced autophagy involves the selective autophagic receptor protein, SQSTM1 and polyubiquitination

**Figure 2.**
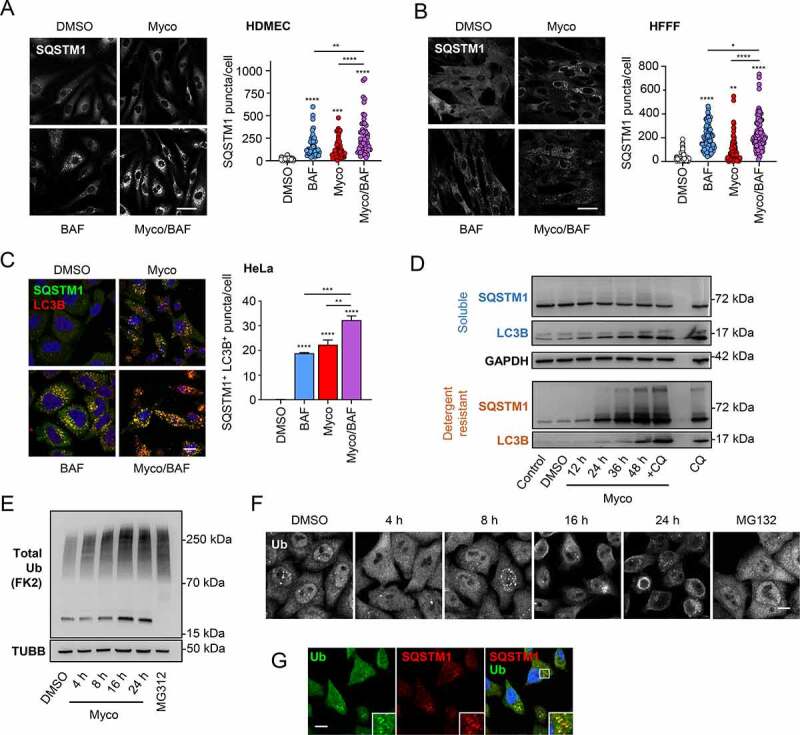
Accumulation of SQSTM1 and ubiquitinated proteins in mycolactone-exposed cells. Cells were exposed to mycolactone (Myco) at the minimal inhibitory concentration, alongside a solvent control (0.05% DMSO, equivalent dilution to the mycolactone) for the longest timepoint tested. Where included, bafilomycin A_1_ (BAF) was used at cell-specific saturating concentrations. All immunofluorescence images were obtained using a Nikon A1 confocal laser scanning microscope. For all immunoblots, the migration relative to known molecular mass markers is shown. For all panels, unless otherwise indicated, statistical comparisons are to the DMSO-treated cells of that particular cell type, ns = not significant; * = *p*< 0.05; ** = *p*< 0.01; *** = p < 0.001; **** = p < 0.0001. (A) HDMEC exposed to 10 ng/ml mycolactone with and without 100 nM BAF for 8 h were fixed with PFA for SQSTM1 staining with a mouse anti-SQSTM1 antibody (scale bar: 50 µm). Values represent the number of puncta detected per cell for 45–100 cells. Data are representative of 2 independent experiments. (B) HFFF exposed to 31.25 ng/ml mycolactone with and without 200 nM BAF for 8 h were fixed with PFA for SQSTM1 staining with a mouse anti-SQSTM1 antibody (scale bar: 50 µm). Values represent the number of puncta detected per cell for 45–100 cells. Data is representative of 2 independent experiments. (C) HeLa cells were exposed to 31.25 ng/ml mycolactone for 24 h, in the presence or absence of 100 nM BAF for the final 12 h, then fixed with methanol for SQSTM1 with a mouse anti-SQSTM1 antibody (green) and LC3B (red) staining. Nuclei were counterstained with DAPI (blue) (scale bar: 10 µm). Values are means of 3 independent experiments ± SEM. (E) HeLa cells were left untreated, exposed to mycolactone for different times (as indicated) or 50 µM chloroquine (CQ) for 12 h. The 48-h timepoint was performed without and with 50 µM CQ (for the final 12 h). Cells were harvested in RIPA lysis buffer, then soluble and insoluble fractions were prepared as described in Methods prior to immunoblotting. Data are representative of 3 independent experiments. (F) HeLa cells exposed to 31.25 ng/ml mycolactone for various times or 10 µM MG132 for 8 h were lysed directly in gel sample buffer prior to immunoblotting. Data are representative of 2 independent experiments. (G) HeLa cells were exposed to 31.25 ng/ml mycolactone for the times indicated, or 10 µM MG132 for 8 h then fixed with PFA for total ubiquitin staining with FK2 (scale bar: 10 µm). Data are representative of 2 independent experiments. (H) HeLa cells were exposed to 31.25 ng/ml mycolactone for 16 h then fixed with PFA for total ubiquitin with FK2 (green) and SQSTM1 with a rabbit anti-SQSTM1 antibody (red) staining (scale bar: 10 µm).

Mycolactone significantly increased the appearance of puncta staining positively for SQSTM1 in both HDMEC and HFFF cells within 8 h (Figure 2A and 2B). This was further increased in the presence of bafilomycin A_1_, indicating that this observation is unlikely to be due to inhibition of lysosomal clearance. In HeLa cells SQSTM1-positive puncta accumulated over time but more slowly and did not reach statistical significance until 16 h after mycolactone addition (**Fig. S2A**).

We next assessed the extent of colocalization between SQSTM1 and LC3B in response to mycolactone. As expected, high levels of colocalization were observed in the presence of bafilomycin A_1_, an effect also elicited by mycolactone alone (Figure 2C **and S2B**). Exposure to both mycolactone and bafilomycin A_1_ gave rise to a further, significant increase in double-positive puncta numbers compared to either compound alone, providing additional evidence for a genuine increase in autophagic flux.

As SQSTM1 aggregates are frequently insoluble [34], we analyzed both detergent-soluble and -resistant fractions of HeLa cells exposed to mycolactone by immunoblotting. Levels of soluble SQSTM1 decreased with longer exposure to mycolactone, while processing of LC3B to LC3B-II increased over time (Figure 2D). By contrast, SQSTM1 levels in detergent-resistant fractions showed a marked increase after 24-h mycolactone exposure (approximately 48 h before these cells die) [16]. Addition of chloroquine to mycolactone-exposed cells prior to lysis caused a further increase of LC3B-II. Similar results were obtained in macrophage-like RAW264.7 and MEF cell lines (**Fig. S2C and S2D**).

To investigate the role played by polyubiquitin in mycolactone-induced selective autophagy we carried out immunoblot analysis of HeLa cells using a well-characterized pan-ubiquitin antibody, FK2. Total levels of polyubiquitin-conjugated proteins were strongly increased, as expected [19], in whole cell lysates following exposure to mycolactone to levels comparable to the positive control, MG132 (Figure 2E). By immunofluorescence, ubiquitin was predominantly nuclear in control cells but shifted progressively to a more cytoplasmic location over time with mycolactone, with large numbers of cytoplasmic puncta detectable after 16–24 h (Figure 2F). While the proteasome inhibitor MG132 caused an increase in overall cytoplasmic staining, this was diffuse, and cells contained relatively few discrete puncta. Co-staining with anti-SQSTM1 antibodies revealed a high degree of overlap with cytoplasmic ubiquitin puncta after 24 h incubation with mycolactone (Figure 2G). A close association between SQSTM1 and ubiquitin was also observed in HFFF (**Fig. S2E**).

These experiments suggest that SQSTM1 accumulation in mycolactone-exposed cells may be a response to the increase in cytosolic ubiquitinated proteins. The enhanced levels of SQSTM1 puncta in the presence of bafilomycin A_1_ suggest that this is dependent on an increased autophagic flux, but protein aggregation and changes in SQSTM1 expression may also contribute to these changes.

### Mycolactone-induced autophagy is dependent on SEC61 inhibition

**Figure 3.**
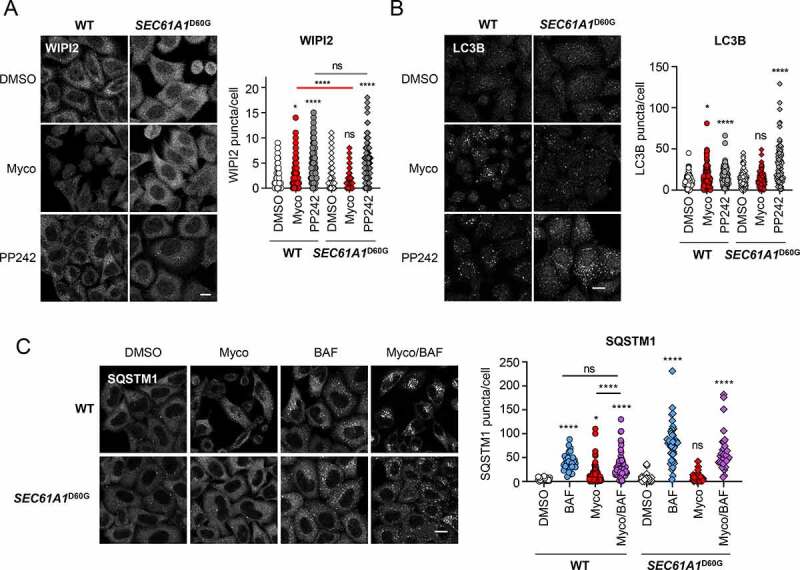
Mycolactone-induced changes in autophagy are dependent on SEC61 inhibition.

To confirm that the increase in autophagy seen after mycolactone exposure was a consequence of SEC61 inhibition, we used stably transfected HeLa cells harboring a missense mutant of *SEC61A1* (*SEC61A1^D60G^*). This is one of a series of mutations in *SEC61A1* found to provide resistance to mycolactone in reverse genetic screening due to reduced mycolactone binding [16,35,36]. The expression of the Flag-tagged mutant protein was confirmed by immunoblotting and immunofluorescence (**Fig. S3A and S3B**) and the HeLa cells were resistant to mycolactone-induced cytotoxicity (**Fig. S3C**). Mycolactone induced formation of both WIPI2 and LC3B puncta in HeLa cells as before; however, this did not occur in cells overexpressing the mutant SEC61A1 (Figure 3A and 3). Conversely, both wild-type and mutant cells exhibited increased WIPI2 and LC3B puncta formation in response to PP242. For SQSTM1, we saw a more muted induction in wild type HeLa than in primary cells, as before (Figure 3C). Here, co-incubation with mycolactone and bafilomycin A_1_ seemed to result in very strong staining for puncta that could potentially be aggregates due to the dual pressure of the different inhibitors. The potential that this may have resulted in undercounting might explain why the absolute number of puncta is lower versus bafilomycin A_1_ alone. However, cells expressing SEC61A1^D60G^ no longer showed an increase in SQSTM1 puncta after exposure to mycolactone (Figure 3C).

Parental (WT) and HeLa cells stably overexpressing mycolactone-resistant *SEC61A1^D60G^* were exposed to 0.05% DMSO, 31.25 ng/ml mycolactone (Myco) and/or 1 μM PP242 for 1 h or 100 nM BAF for 4 h. All immunofluorescence images were obtained using a Nikon A1 confocal laser scanning microscope (scale bar: 10 μm). For all panels, unless otherwise indicated, statistical comparisons are to the DMSO-treated cells of that particular cell type; ns = not significant; * = *p*< 0.05; ** = *p* < 0.01; *** = p < 0.001; **** = p < 0.0001. (**A**) Cells were exposed to mycolactone for 8 h or 1 μm PP242 for 1 h and fixed and stained for WIPI2. Values represent the number of puncta detected per cell for at least 140 cells. Data are representative of 3 independent experiments. (**B**) Cells were exposed to mycolactone for 16 h or PP242 then fixed with methanol for LC3B staining. Values represent the number of puncta detected per cell for at least 128 cells. Data is representative of 2 independent experiments. (**C**) Cells were exposed to mycolactone for 16 h with or without BAF, then fixed with PFA for SQSTM1 staining with a mouse anti-SQSTM1 antibody. Values represent the puncta detected per cell for 30–100 cells per treatment. Data are representative of 3 independent experiments.

Of the ATG proteins, only one has transmembrane domains, ATG9A, making it a potential target of SEC61 inhibition. However, ATG9A is a multipass membrane protein and these are generally insensitive to the inhibitory effects of mycolactone [17]. Instead, mycolactone primarily targets type I and II single-pass membrane and secreted proteins [9,10]. Hence, these effects of mycolactone cannot be explained by changes in ATG9A levels as mycolactone appears to have little effect on ATG9A expression in HFFF cells (**Fig. S3D**). Furthermore, mycolactone induces a dispersal of ATG9A from its perinuclear location, in a manner similar to that reported for mammalian cells subjected to starvation or treated with rapamycin [37] (**Fig. S3E**).

Taken together the data suggest the responses we see are driven by blocking of translocation of newly synthesized proteins into the ER which leads to a subsequent buildup of mislocalized, ubiquitinated proteins in the cytoplasm that in turn associate with SQSTM1 and LC3B. The secondary nature of the response to mycolactone may explain the slower appearance of early autophagy markers such as WIPI2 when compared to direct inhibitors of autophagy regulating enzymes such as PP242, or acute nutrient starvation.

### Expression of SQSTM1 protects against mycolactone cytotoxicity but is not required for increased autophagy initiation

**Figure 4.**
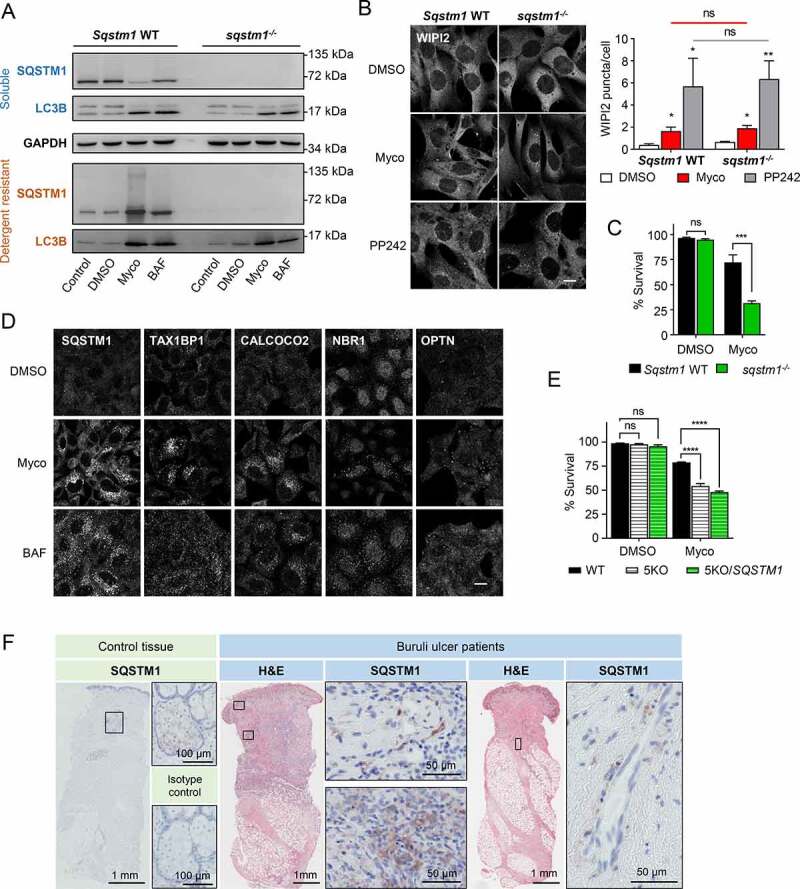
Increased expression of SQSTM1 enhances cell survival in the presence of mycolactone but is not required for initiation of autophagy. Cells were exposed to 31.25 ng/ml mycolactone (Myco), alongside a solvent control (0.05% DMSO, equivalent dilution to the mycolactone) for the longest timepoint tested. All immunofluorescence images were obtained using a Nikon A1 confocal laser scanning microscope (scale bar: 10 µm). For all immunoblots, the migration relative to known molecular mass markers is shown. For all panels, unless otherwise indicated, statistical comparisons are to the DMSO-treated cells of that particular cell type, ns = not significant; * = *p*< 0.05; ** = *p*< 0.01; *** = p < 0.001; **** = p < 0.0001.(A-C) Matched *Sqstm1* WT and *sqstm1^−/-^* MEFs were exposed to mycolactone for the indicated times. As controls, either 100 nM bafilomycin A_1_ (BAF) for 12 h or 1 µM PP242 for 1 h were used, as indicated. (A) Cells were harvested in RIPA lysis buffer, then soluble and insoluble fractions were prepared as described in Methods prior to immunoblotting. Migration relative to known molecular mass markers is shown. Twelve-hour mycolactone exposure. Data are representative of 3 independent experiments. (B) Cells were fixed and stained for WIPI2 after 8 h mycolactone exposure. Values represent the means of 3 independent experiments ± SEM. (C) Cell viability in *Sqstm1* WT and *sqstm1^−/-^* MEF as measured by CellEvent assay after 24 h mycolactone exposure. Values represent the mean of 3 independent experiments ± SEM. (D) WT HeLa cells exposed to mycolactone for 16 h before fixing with PFA for immunofluorescent staining with mouse anti-SQSTM1, anti-TAX1BP1, anti-CALCOCO2, anti-NBR1 or anti-OPTN antibodies. Data are representative of duplicate experiments. (E) Cell viability for parental HeLa (WT), PentaKO cells (5KO) and PentaKO cells with reconstituted SQSTM1 expression (5KO/*SQSTM1*) as measured by CellEvent assay after 48 h mycolactone exposure. Values represent the mean of 3 independent experiments ± SEM. (F) Skin sections from samples from healthy donor (left hand panel) and Buruli ulcer patient lesions (right hand panels), stained with anti-SQSTM1 antibody or isotype-matched control. Boxes show locations of magnified SQSTM1-stained sections. Comparable results were found for all 8 patient biopsies analyzed regardless of whether the lesion was an ulcer or a plaque.

Next, we sought to characterize the role played by SQSTM1 in mycolactone-induced cytotoxicity by comparing the response between *Sqstm1* WT and *sqstm1^−/-^* MEFs [34,38]. Immunoblotting confirmed SQSTM1 deletion in knockout cells, while mycolactone induced a shift of SQSTM1 into detergent-resistant fractions in matched WT cells (Figure 4A). Expression of SQSTM1 was not required for LC3B processing because similar levels of LC3B-II could be seen in both *Sqstm1* WT and *sqstm1^−/-^* cells after mycolactone exposure (Figure 4A). Similarly, both *Sqstm1* WT and *sqstm1^−/-^* MEFs exhibited an increase in WIPI2 puncta, showing that SQSTM1 itself is not required for initiation of autophagy triggered by SEC61 inhibition or MTOR inactivation by PP242 (Figure 4B). This is unlikely to be due to other ubiquitin-binding receptor proteins taking the place of SQSTM1 in the knockout MEF cells since CALCOCO2, NBR1, TAX1BP1 and OPTN all showed a limited response to mycolactone compared to SQSTM1 in both *Sqstm1* WT and *sqstm1^−/-^* cells (**Fig. S4A**).

Both pro-survival and pro-apoptotic roles have been demonstrated for SQSTM1; however, given its involvement in eliminating toxic protein aggregates and restoring cellular homeostasis, we hypothesized that elevated SQSTM1 could be an adaptive response to the increased cytosolic (polyubiquitinated) protein burden, rather than a contributing factor to mycolactone’s cytotoxicity. To address this question, we assessed the viability of *Sqstm1* WT and SQSTM1-deficient MEFs after mycolactone exposure. Here, *sqstm1^−/-^* MEFs showed a significantly accelerated loss of viability compared to their *Sqstm1* WT counterparts (Figure 4C **and S4B**). From these data, we conclude that SQSTM1 is protective against, rather than a driver of, mycolactone’s cytotoxicity.

To investigate the relative role of SQSTM1 in the cellular response to mycolactone in comparison with other mammalian autophagy receptors, we used HeLa cells in which expression of the five human receptors (SQSTM1, TAX1BP1, NBR1, CALCOCO2 and OPTN) had been ablated by gene editing [39], known as PentaKO (5KO) cells. In WT HeLa cells, all receptors except OPTN showed some increase in puncta in the presence of mycolactone (Figure 4D). As previously reported [16], WT HeLa cells exposed to mycolactone exhibit a slower rate of death than MEFs. However, in keeping with the viability assays on *sqstm1^−/-^* MEFs, the death rate induced by mycolactone was accelerated in PentaKO cells (**Fig. S4C**). Immunofluorescence analysis revealed a general increase in cytosolic ubiquitin in PentaKO cells, but in contrast to WT HeLa cells, this was diffuse and did not localize to discrete puncta (**Fig. S4D**), confirming that receptor proteins are needed for this step. Interestingly, reconstitution of SQSTM1 expression (**Fig. S4E**) failed to restore viability (Figure 4E). Thus, although SQSTM1 plays an important role in cell survival, baseline levels of expression are insufficient to protect the cells from the buildup of mislocalized proteins and upregulation of expression may also be a requirement.

Since mycolactone is the pathogenic mediator of BU, we used immunohistochemistry to determine whether SQSTM1 expression changes in BU lesions (**Table S1**). In normal human skin, very little SQSTM1 staining was detected, except for a relatively weak signal seen close to sebaceous glands (Figure 4F, left-hand panel). In biopsies taken from the infected skin of BU patients, their lesions contained necrotic regions (N), which display features of coagulative necrosis and non-necrotic regions which include infiltrated regions containing inflammatory cells. Focusing on the non-necrotic regions, we observed SQSTM1 staining in all eight lesions examined, particularly in areas displaying heavy infiltration of inflammatory cells (Figure 4F). The levels varied between region and cell type; in some areas, SQSTM1 expression was quite widespread while, in others, strong staining was observed in individual cells (Figure 4F, middle panel). In two patients, SQSTM1 was detected close to blood vessels in sub-endothelial regions, associated with long, spindle-shaped cells, which might be smooth muscle cells (Figure 4F right-hand panel). This suggests that the upregulation in SQSTM1 protein expression in response to mycolactone also occurs *in vivo*.

### Mycolactone induction of autophagy requires RB1CC1 but is ULK-independent

**Figure 5.**
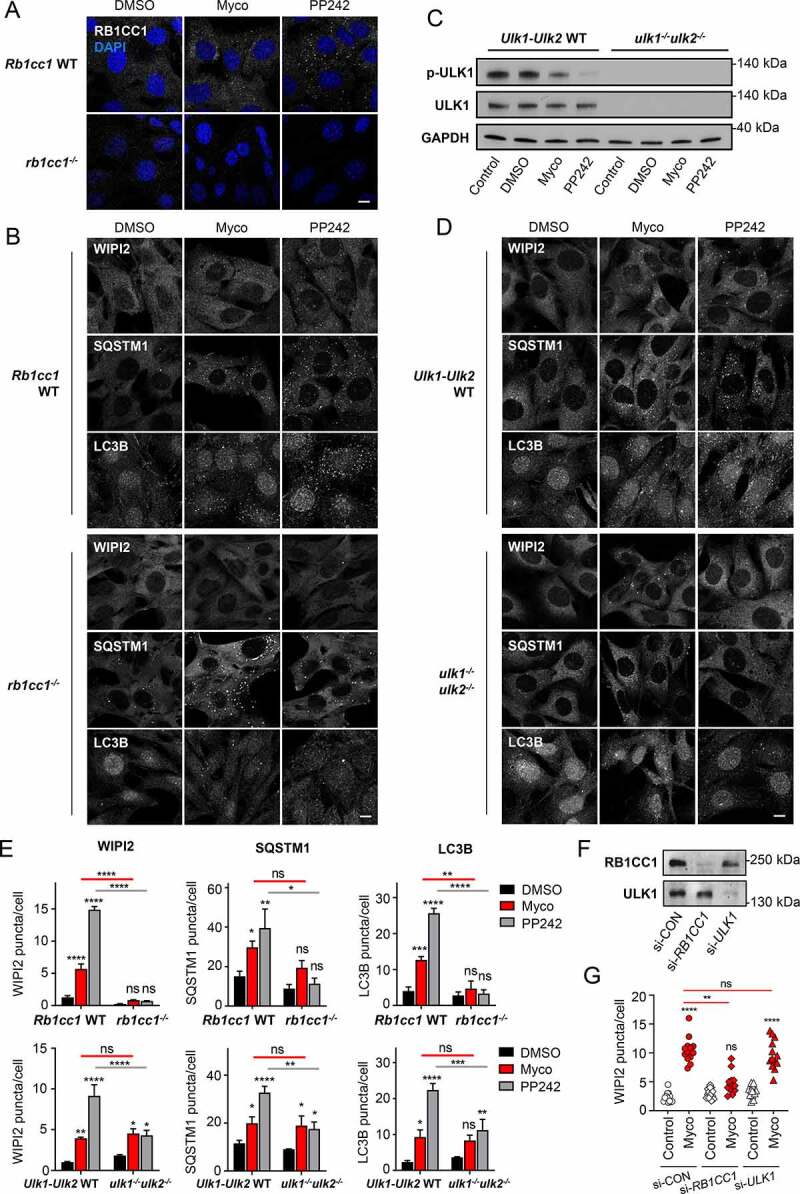
Mycolactone stimulates a non-canonical pathway of autophagosome biogenesis.

Key proteins in the formation of the phagophore are the members of the ULK-complex; RB1CC1 and ULK1/2. In order to dissect the mechanism of autophagy induced by mycolactone, we examined the role of these proteins using knockout MEFs. As observed in HDMEC and HFFF, exposure of *Rbcc1* WT MEFs to mycolactone for 8 h led to an increase in RB1CC1-positive puncta (Figure 5A **and S5A**). In cells lacking RB1CC1, not only were there no RB1CC1 puncta detected (**Fig. S5A**), but WIPI2 puncta were also drastically reduced in both mycolactone-exposed and PP242-exposed cells; this was also true for LC3B puncta (Figure 5B and 5E). Although RB1CC1 deficiency caused a reduction in SQSTM1-labeled bodies, these were not significantly reduced in mycolactone exposed cells. The area of the SQSTM1 puncta was, however, generally larger than that seen in control cells (Figure 5B and S5B). Hence, the early stages of autophagosome formation following mycolactone exposure are dependent on RB1CC1, while condensation of SQSTM1 aggregates is not. In line with this, survival of RB1CC1-deficient cells was significantly reduced compared to the parental wild-type MEF cells (**Fig. S5C**) confirming an important protective role for autophagy in cells exposed to mycolactone.

(**A-E**) Matched parental *Rb1cc1* WT and *rb1cc1*^−/-^ MEFs, or matched parental *Ulk1-Ulk2* WT and *ulk1^−/-^ulk2^−/-^* MEFs (as indicated) were exposed to 31.25 ng/ml mycolactone (Myco) for 8 h alongside a solvent control (0.05% DMSO, equivalent dilution to the mycolactone) for the longest timepoint tested. When needed 1 µM PP242 for 1 h was used as a control. All immunofluorescence images were obtained using a Nikon A1 confocal laser scanning microscope (scale bar: 10 µm). For all panels, unless otherwise indicated, statistical comparisons are to the DMSO-treated cells of that particular cell type, ns = not significant; * = *p*< 0.05; ** = *p* < 0.01; *** = p < 0.001; **** = p < 0.0001. Results are representative of 3 independent experiments (**A-D**) or the mean ± SEM of 3 independent experiments (**E**).(**A**) Cells were fixed and stained for RB1CC1. (**B** and **D**) Cells were fixed with PFA for WIPI2 and SQSTM1 staining or fixed with methanol for LC3B staining. (**C**) Cells were lysed directly in gel sample buffer prior to immunoblotting. Migration relative to known molecular mass markers is shown. (**E**) Quantification of WIPI2, SQSTM1 and LC3B puncta for cells shown in B and D. Values represent the means of 3 independent experiments ± SEM. (**F** and **G**) HFFF cells were double transfected with non-targeting (ctrl), *RB1CC1* or *ULK1* SMARTpool siRNA oligonucleotides for a total of 60 h. (**F**) Cells were lysed directly in gel sample buffer prior to immunoblotting. Migration relative to known molecular mass markers is shown. (**G**) Cells were exposed to mycolactone for 6.5 h then fixed for WIPI2 staining. Values represent the mean puncta per cell for 13 fields containing at least 60 cells.

Concerning the role of ULK1 and ULK2, we first examined the phosphorylation of ULK1 in *Ulk1-Ulk2* WT MEF. As expected, the MTOR inhibitor PP242 caused rapid dephosphorylation of ULK1 at Ser727 within 1 h. In contrast, an 8 h exposure to mycolactone reduced ULK1 phosphorylation to a much smaller extent, which did not reach statistical significance (Figure 5C **and S5D**). As expected [40], no ULK1 protein was detected in the double knockout (Figure 5C). However, despite the slightly reduced levels of ULK1 phosphorylation in mycolactone-exposed cells, the absence of the ULK kinases had no impact on the increase in autophagy initiation by mycolactone, as assessed by the appearance of WIPI2, SQSTM1 and LC3B puncta (Figure 5D and 5E). By contrast, the response to PP242 was reduced by approximately 50% for each of these proteins. This suggests that ULK1 and/or ULK2 are not involved in the early stages of the mycolactone-dependent increase in autophagy initiation; accordingly, cells lacking both ULK kinases died at the same rate as their parental wild-type cells (**Fig. S5E**).

These findings were replicated in primary HFFF cells using an siRNA knockdown approach. Knockdown of *RB1CC1* (Figure 5F) caused a significant reduction in WIPI2 puncta in mycolactone-exposed cells (Figure 5G). By contrast, loss of *ULK1* had no effect (Figure 5F and 5G). This supports the conclusion that the response to mycolactone is dependent on RB1CC1 but not ULK kinases. This surprising finding led us to explore potential mechanisms that might regulate this unusual autophagic response.

### SQSTM1-dependent selective autophagy induced by mycolactone is controlled at the level of translation

**Figure 6.**
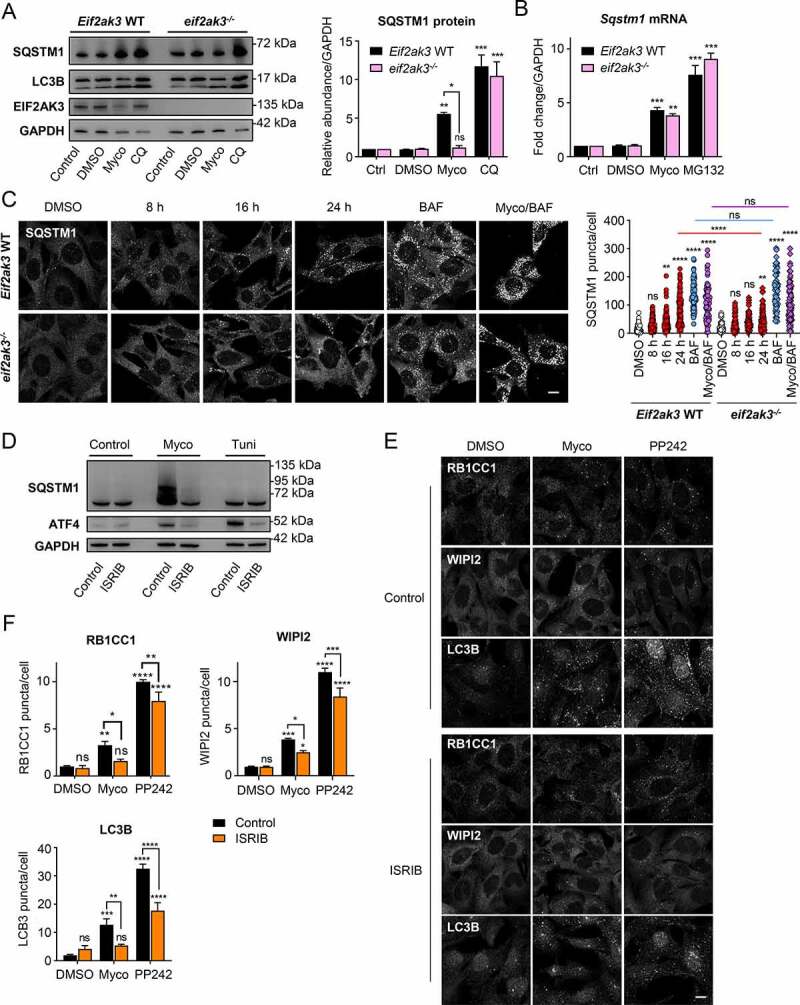
Mycolactone-induced autophagy markers are dependent on the integrated stress response.

Since baseline SQSTM1 expression was not sufficient to protect PentaKO cells from mycolactone exposure (Figure 4E), we considered whether regulation of its expression was important. In our previously published data set for translational profiling of mycolactone-treated cells, the *Sqstm1* mRNA showed a 1.8-fold increase in translational efficiency after mycolactone exposure compared to control cells (p = 0.004) [16]. Hence, it seemed possible that the mycolactone-induced ISR was involved in the regulation of SQSTM1 expression. In MEFs lacking the EIF2S1 kinase EIF2AK3 [16], resting levels of SQSTM1 were equivalent to WT cells but levels of SQSTM1 protein were no longer increased after exposure to mycolactone (Figure 6A). However, the lack of EIF2AK3 had less impact on mycolactone-dependent LC3B processing and the accumulation of SQSTM1 in response to chloroquine was not affected. Similar results were obtained when using bafilomycin A_1_ as a control (**Fig. S6A**). A small increase in the steady state level of *Sqstm1* mRNA in mycolactone exposed cells was not affected by the absence of EIF2AK3 (Figure 6B), hence the increased expression of SQSTM1 appears to be regulated post-transcriptionally. To rule out transcriptional regulation of SQSTM1 by NFE2L2/Nrf2 (nuclear factor, erythroid 2 like 2) following mycolactone exposure, we quantitated expression of the NFE2L2 target *Osgin1* (oxidative stress induced growth inhibitor 1) [41,42]. While the NFE2L2 activator monomethyl fumarate efficiently induced an increase in steady state *Osgin1* expression in both *Eif2ak3* WT and *eif2ak3^−/-^* MEFs, mycolactone had no effect at either 6 or 24 h (**Fig. S6B**).

Cells were exposed to 31.25 ng/ml mycolactone (Myco), alongside a solvent control (0.05% DMSO, equivalent dilution to the mycolactone) for the longest timepoint tested. For all immunoblots, the migration relative to known molecular mass markers is shown. All immunofluorescence images were obtained using a Nikon A1 confocal laser scanning microscope (scale bar: 10 µm). For all panels, unless otherwise indicated, statistical comparisons are to the DMSO-treated cells of that particular cell type, ns = not significant; * = *p* < 0.05; ** = *p* < 0.01; *** = p < 0.001; **** = p < 0.0001. (**A-C**) Matched *Eif2ak3* WT or *eif2ak3^−/-^* MEFs were left exposed to mycolactone for 24 h (or the times indicated). Either 50 µM chloroquine (CQ) 12 h or 10 μM MG132 for 16 h were used as controls. Where co-incubated, CQ was included for the final 12 h. Values represent the mean of 3 independent experiments ± SEM. (**A**) Cells were lysed directly in gel sample buffer prior to immunoblotting. Quantification of SQSTM1 abundance represents pixel intensity relative to GAPDH controls. (**B**) Total RNA was extracted from cells and subjected to qRT-PCR; data were normalized to *Gapdh* mRNA. (**C**) Cells were exposed to mycolactone for the times indicated, with or without 100 nM BAF for 4 h then fixed with PFA for SQSTM1 staining. Values represent the number of puncta in each cell for 60–100 cells ± SD. Data are representative of 2 independent experiments. (**D**) HeLa cells were exposed mycolactone for 24 h, or 5 µg/ml tunicamycin (Tuni) for 6 h, in the presence or absence of 100 nM ISRIB then lysed directly in gel sample buffer. Data are representative of 3 independent experiments. (**E** and **F**) WT MEF were exposed to mycolactone for 8 h or 1 µM PP242 for 1 h in the presence or absence of 200 nM ISRIB. Cells were fixed with PFA for WIPI2 and RB1CC1 staining, or with methanol for LC3B staining. (**F**) Quantification of puncta/cell for WIPI2, RB1CC1 and LC3B. Values represent the mean of triplicate experiments ± SEM.

Immunofluorescence staining for SQSTM1 in WT MEFs showed a time-dependent increase of SQSTM1 puncta in response to mycolactone similar to that seen in HeLa cells (Figure 6C); however, these changes were markedly impaired in EIF2AK3-deficient cells. Moreover, there was no difference between these cell lines in number of SQSTM1 puncta observed in response to bafilomycin A_1_ (Figure 6C).

In the absence of EIF2AK3, other EIF2S1 kinases are still able to contribute to the mycolactone-induced ISR [16]. Hence, we also asked if the small molecule ISR inhibitor (ISRIB) could prevent the upregulation of SQSTM1. ISRIB reverses the effects of EIF2S1 phosphorylation by enhancing the guanine nucleotide exchange factor activity of the EIF2B complex [43], enabling the rapid formation of the 43S ternary complex required for efficient translation initiation even in the presence of p-EIF2S1. In HeLa cells, both mycolactone and the ISR inducer, tunicamycin, efficiently promoted ATF4 translation as previously reported [16,44], and this was completely reversed by ISRIB (Figure 6D). ISRIB also completely reversed the upregulation of SQSTM1 elicited by mycolactone, providing additional evidence that EIF2S1 phosphorylation is required for the translational control of SQSTM1.

To investigate the role of the ISR in the development of the early autophagic response, WT MEF were incubated with mycolactone in the presence or absence of ISRIB at a concentration that blocked ATF4 induction in these cells (**Fig. S6C**). While the effect was not as marked as that seen in RB1CC1-deficient cells, numbers of RB1CC1, WIPI2 and LC3B puncta were all reduced in the presence of ISRIB (Figure 6E and 6F). Interestingly, the response to PP242 was also reduced, particularly with respect to the increase in LC3B-positive puncta. This last effect was reproduced in MEFs expressing a non-phosphorylatable mutant of EIF2S1, EIF2S1^S51A^ (**Fig. S6D and S6E**). These cells were also highly sensitive to mycolactone, and approximately 70% were dead after 24 h exposure (**Fig. S6F**). Taken together, the results show that the ISR is critical to the RB1CC1-dependent, ULK-independent pathway of protective autophagy that is by induced by mycolactone.

## Discussion

The role of selective autophagy in managing misfolded or aggregated proteins in the cytosol is increasingly being recognized as a key cellular process contributing to several clinically important diseases, including cancer [30,45,46], neurodegenerative diseases [47–51] and now, BU. We have identified an adaptive response to SEC61 inhibition by mycolactone involving some, but not all, components of the classical autophagy pathway and the ISR-driven translational upregulation of the autophagic receptor protein SQSTM1. These combine with the UPS to eliminate mislocalized/misfolded proteins in the cytosol, prolonging cellular survival in the presence of the toxin.

A role for autophagy in BU is backed up by recent genetic evidence such as a case-control genome association study of BU patients that reported variants in the genes encoding three different autophagy-related proteins that are linked to disease susceptibility: *NOD2* (nucleotide-binding oligomerization domain-containing protein 2), *PRKN* (parkin RBR E3 ubiquitin protein ligase) and *ATG16L1* [52]. While the single nucleotide polymorphism rs1333955 in *PRKN* correlates with a generally increased risk of BU disease, the *NOD2* variants rs9302752 and rs2066842 may specifically contribute to BU severity, being more commonly associated with severe disease [52]. By contrast, the rs2241880 single nucleotide polymorphism in *ATG16L1* confers a protective effect against ulceration, a finding replicated in a genome-wide association study [52,53]. This variant has been associated with decreased autophagy activation resulting from CASP3 (caspase 3)-CASP7 degradation of the ATG16L1 protein [54]. The same polymorphism is also associated with decreased selective autophagy, accumulation of SQSTM1 and higher levels of pro-inflammatory IL1B (interleukin 1 beta/IL-1β) *in vitro* [54]. It is not yet known whether these mutations have a direct impact on the cellular response to mycolactone or alter the global host response to the bacteria themselves. However, the interaction between ATG16L1 and apoptotic pathways is intriguing as several studies have shown that the ultimate cause of cell death in mycolactone exposed cells is apoptosis, most recently in [55], where a range of different inhibitors of various cell death pathways were tested and only those blocking apoptosis had a protective effect. Notably, IL1B has very recently been shown to be present in the skin following *M. ulcerans* infection due to the action of mycolactone-containing vesicles [56].

Our investigation of BU patient skin biopsies suggests that the effects of mycolactone on cultured cells may be relevant to the host response during infection. Anti-SQSTM1 immunohistochemistry revealed positive staining in regions of the lesions that had been infiltrated by immune cells such as monocytes. While the *M. ulcerans* bacilli are typically found only at the base of the subcutis [57], it is known that cells distant from the bacteria can be affected by mycolactone that has diffused away from the bacteria due to its lipid-like nature. Mycolactone is generally considered to be anti-inflammatory due the macroscopic appearance of Buruli ulcer, and mycolactone’s strong inhibitory effect on the production of SEC61-dependent cytokines and chemokines, as well as immune cell surface receptors [7,11,58]. However, the recent finding that BU lesions contain IL1B may necessitate some reassessment of these assumptions [56]. Hence, there are currently two scenarios that could explain the accumulation of SQSTM1 in BU lesions. Either it is secondary to inflammation due to IL1B or it is a direct consequence of mycolactone initiating an increase in cellular SQSTM1 levels. Since the pathway we have described here is not dependent on cells retaining SEC61-dependent cytokine receptors, we propose that this may be the most likely explanation. The involvement of autophagy in BU warrants further investigation, particularly considering recent reports of autophagy’s role in revascularization and wound healing of cutaneous lesions [59–61].

Although others have previously linked mycolactone and autophagy by investigating LC3B processing induced by the toxin, Gama *et al*. concluded that mycolactone blocks the final stages of autophagy, based on experiments demonstrating no additional increase in LC3B-II abundance in HeLa cells after combined exposure to bafilomycin A_1_ [19]. This discrepancy with our findings may be due to their use of detergent soluble fractions, which only detects a portion of the total LC3B-II present in the cell (See Figure 2D). Our conclusion that mycolactone increases autophagic flux is based on the use of a number of different approaches including immunoblotting, immunofluorescence and tandem-tag labeling. Increased WIPI2, RB1CC1 and ATG16L1 puncta formation is indicative of an increase in autophagic initiation, while increases in SQSTM1 and LC3B puncta counts upon exposure to mycolactone and bafilomycin A_1_ suggest increased autophagic flux. These observations point to a genuine induction of the pathway rather than a simple block at the final degradation stage. However, it should be noted that simultaneous increases in autophagy induction and late-stage inhibition has been reported for the Alzheimer disease drug C10 [62], and we cannot yet completely rule out a similar “dual effect” for mycolactone.

The autophagic response to mycolactone is dependent on its ability to inhibit protein translocation into the ER: we were unable to detect any discernible effect of mycolactone on WIPI2, LC3B or SQSTM1 puncta formation in cells expressing a mutant of *SEC61A1* (*SEC61A1^D60G^*) that is resistant to the cytotoxic effects of mycolactone. It has previously been shown that inhibition of the cotranslational translocation of newly synthesized proteins into the ER leads to a buildup of proteins intended for export in the cytosol [7]. Hence the proteasome plays an important part in clearing these proteins [6,7], but our findings suggest that selective autophagy may also be needed to protect the cells from the toxic effects of the accumulation of mislocalized proteins once the proteasome becomes overwhelmed. This is the likely explanation for why cells lacking key components of that pathway die more rapidly in the presence of mycolactone.

Upregulation of SQSTM1 can be identified in previously published datasets of genome-wide proteomes and translatomes in a variety of cell lines exposed to mycolactone [13,16,17]. Our work validates those findings and indicates a specific role for the selective autophagy pathway in the cellular response. While there is good evidence that SQSTM1 plays an important role in the sequestration and degradation stages of mycolactone-induced autophagy, it appears not to be required for its initiation. Hence MEFs lacking SQSTM1 clearly showed both increased formation of WIPI2 puncta and upregulation of LC3B-II after mycolactone exposure. None of the other ubiquitin-binding proteins that can act as a scaffold for selective autophagy: OPTN [30], CALCOCO2 [27,29], NBR1 [28] and TAX1BP1 [29], appeared to show an increase in puncta comparable to that seen with SQSTM1, suggesting that there is limited redundancy and it seems unlikely that one or other are taking the place of SQSTM1 in the knockout cell line. Thus, the increased rate of cell death of knockout cells in the presence of mycolactone points to a specific role for SQSTM1. Although SQSTM1 has been implicated in autophagy-mediated cell death and the switch between apoptosis and necroptosis [63], such pathways are not likely to explain this phenotype, since necroptosis inhibitors cannot prevent mycolactone-induced cell death [54]. Whether the prime protective function of SQSTM1 lies in simply its ability to contain the buildup of excess ubiquitinated proteins by sequestering them in insoluble inclusions, as seen in cells exposed to proteasome inhibitors [64], or in selective targeting those proteins to the autophagic pathway is unclear. Co-incubation of mycolactone with bafilomycin A_1_ caused enhanced SQSTM1 puncta detection in primary cells, and colocalization of SQSTM1 with LC3B-II was increased in the presence of bafilomycin A_1_, suggesting that SQSTM1 turnover does occur. However, in HeLa cells, the addition of chloroquine or bafilomycin A_1_ had much less effect on the accumulation of total or insoluble SQSTM1 than on LC3B-II levels, implying a lack of extensive degradation. While this could be explained by the overall more muted autophagy response in these highly transformed cells, it could also indicate that there is some inhibition of autophagy at later stages, similar to that caused by C10 [62]. Hence, both processes may occur in mycolactone-exposed cells and contribute to the protective effect of SQSTM1. Although impairment of autophagy through knockdown of RB1CC1 is detrimental, suggesting the initiation of autophagy following inhibition of SEC61 is important to survival, the sequestration of mislocalized protein aggregates by SQSTM1 may in itself be enough to maintain a degree of cellular homeostasis. Deciphering the relative roles of the different aspects of SQSTM1 function is further complicated by the coincident increased transcription and translation of *Sqstm1* mRNA.

We have previously shown that mycolactone-resistant cells (expressing *SEC61A1*^D60G^) show no increase in ATF4 protein expression when incubated with mycolactone [16]. Furthermore, cells with a reduced capacity to phosphorylate EIF2S1 (*eif2ak3^−/-^ eif2ak4*^−/-^) show no upregulation of LC3B-II [16]. In the current work, inhibition of the ISR with ISRIB was able to reverse the upregulation of SQSTM1 in mycolactone-exposed HeLa cells and the appearance of WIPI2, RB1CC1 and LC3B-II was muted in the presence of the inhibitor. These experiments implicate EIF2S1 phosphorylation as a central control point in the regulation of selective autophagy downstream of SEC61 inhibition. Translational regulation may be essential for the maintenance of SQSTM1 levels when faced with an increase in autophagic flux combined with global translational arrest. This could explain the inability of exogenous SQSTM1 to rescue viability in PentaKO cells. Others have also reported overlap of UPR and SQSTM1-dependent autophagy via EIF2S1 phosphorylation. Such regulation of SQSTM1 was proposed to occur by an “ATF4-like mechanism” by Riz *et al*., who demonstrated increased pro-survival autophagy in carfilzomib-resistant multiple myeloma cell lines as a direct result of EIF2AK3-mediated EIF2S1 phosphorylation, leading to upregulated SQSTM1 translation [45].

Transcription of a number of autophagy-related genes, including SQSTM1, has been shown to be induced via p-EIF2S1-mediated activation of the ATF4/C/EBP homologous protein pathway [65] in nutrient-starved cells and in enterocytes infected with adherent-invasive *E. coli* [66]. However, although a small increase in *Sqstm1* mRNA levels was detected in mycolactone-exposed cells, this was not affected by deletion of EIF2AK3. Expression of SQSTM1 can also be induced by NFE2L2 but we have excluded a role for activation of this pathway in *Eif2ak3* WT or *eif2ak3^−/-^* cells. Interestingly, in a recent report of ER stress-induced autophagy by tunicamycin, the role of EIF2AK3 was shown to be distinct from that of ATF4 and did not appear to involve transcription [67]. Crosstalk between the ISR and autophagy may therefore occur in many ways and at many stages of the process and does not rely on transcriptional changes alone. Although our data strongly support translational control as an important component of SQSTM1 regulation, a role for post-translational modifications such as phosphorylation and ubiquitination cannot be ruled out.

To our knowledge, the current work is the first report suggesting a direct link between WIPI2 puncta formation and translational control by the ISR. The role of the ISR in regulating the autophagic response to mycolactone explains a paradox highlighted in our previous work whereby cells lacking ATF4 expression die at a slower rate but targeting its upstream effectors through knockout of the EIF2S1 kinases EIF2AK3 or EIF2AK4/GCN2, or by overexpression of the EIF2S1 phosphatase PPP1R15A, leads to accelerated cell death [16], as impaired clearance of ubiquitinated proteins in these cells would have a negative impact on survival.

Our results show the ULK kinases to be redundant in the increase in mycolactone-induced autophagy initiation while RB1CC1 is essential. In addition, cells lacking RB1CC1 died much more rapidly than controls, whereas the loss of ULK1 and ULK2 had no impact, reinforcing the relative contribution of these genes. Much of our knowledge of the “canonical” MTORC1- and AMPK-regulated, ULK-dependent pathway of macroautophagy activation comes from studies of cells under conditions of nutrient deprivation or MTORC1 inhibition, but ULKs are not always essential in the induction of autophagy. It is becoming increasingly clear that ULK-independent pathways play an important role in the mammalian cell autophagic response to cellular stressors such as ammonia [68], glucose deprivation [69], hypoxia [70] and ivermectin [71]. RB1CC1 has been shown to interact directly with SQSTM1 via its CLAW domain to facilitate degradation of ubiquitinated cargo and to bind to the ER protein CCPG1 (cell cycle progression 1) to promote reticulophagy [72,73]. Interaction between RB1CC1 and ATG16L is essential for ULK-dependent but dispensable for ULK-independent autophagy induction [69]. Therefore, direct interactions between RB1CC1 and other autophagy pathways proteins may be driving the increase in autophagic flux in our system. Interestingly, coibamide A, a depsipeptide marine cyanobacterium toxin, which has recently been shown to inhibit SEC61 in a manner similar to mycolactone, also induces an ULK-independent pathway of autophagy [74,75]. SEC61 inhibitors may therefore prove useful tools in dissecting these autophagy pathways that rely on a subset of autophagy proteins.

This work highlights the interplay between the ISR and autophagy and indicates that in addition to the established transcriptional upregulation of autophagy mediators [65], some may also be directly regulated at the post-transcriptional level. In the case of mycolactone, as toxic mislocalized proteins accumulate, cells must balance the cessation of global protein production by ISR-driven translational arrest with the need to increase expression of proteins essential for restoring homeostasis through the ISR and autophagy. Future work establishing the role of proteins such as ATG16L1 in disease progression and determination of the true nature of the SQSTM1 puncta by proteomics and correlative electron microscopy, would allow us to unravel the precise mechanisms by which they achieve this and could be important for our understanding of not just BU pathology, but also other disease processes where selective autophagy is implicated, such as cancer and neurodegeneration.

## Materials and methods

### Reagents and cell culture

For all experiments, we used synthetic mycolactone A/B [76], which was generously donated by Prof. Yoshito Kishi (Harvard University). HeLa cells were obtained from ATCC (CCL-2). PentaKO cells were a gift from Prof. Michael Lazarou (Monash University, Australia) [39]. Human Caucasian fetal foreskin fibroblast cells (HFFF2) were purchased from Merck (86031405). HDMEC were from Promocell (C-12,210). *eif2ak3^−/-^, Eif2s1^S51A^*, and *sqstm1^−/-^* and respective WT MEFs have been described previously [34,38,77,78] and were kindly donated by Prof. Ronald Wek (Indiana University), Dr Mark Caldwell (University of Southampton) and Prof. Terje Johansen (Arctic University of Norway), respectively. *Rb1cc1* WT and *rb1cc1^−/-^* MEFs [79] were a gift from Prof Jun Lin Guan (University of Cincinnati) while *Ulk1-Ulk2* WT and *ulk*1^−/-^*ulk2^−/-^* double-knockout MEFs [40] were from Sharon Tooze (The Francis Crick Institute, London). All cells except HDMEC were grown at 37°C, 5% CO_2_ in high-glucose DMEM (Merck, D6429) supplemented with 10% FBS (Gibco, 10500064). In the case of WT and mutant MEF cell lines and PentaKO HeLa cells, cells were additionally supplemented with 1 mM non-essential amino acids (Gibco, 11140035) 50 µM β-mercaptoethanol (Gibco, 31350010) and 100 µg/ml penicillin/streptomycin (Gibco, 5140122). HDMEC were cultured at 37°C, 5% CO_2_ in Endothelial Cell Growth Medium MV (Promocell, C22120).

Mycolactone was diluted from a 500 µg/ml stock in DMSO (Merck, D2560) and was used at the minimal inhibitory concentration in all experiments. This was usually 31.3 ng/ml (~42 nM) in all cells except for HDMEC, which are more susceptible to mycolactone and therefore require only 10 ng/ml (~13 nM) mycolactone for maximal effect [6]. Chloroquine (Merck, C6628) was diluted to 50 µM from a stock in sterile ultrapure water and bafilomycin A_1_ (Invivogen, tlrl-baf1) was used at 100–200 nM, diluted from a DMSO stock. PP242 (Bio-Techne Ltd, 4257) was used at 1 µM. MG132 (Merck, M8699) was used at a final concentration of 10 µM and monomethyl fumarate (Merck, 651419) at 100 µM. ISRIB, diluted from a DMSO stock (Merck, SML0843), was used at 100 nM for HeLa cells and HDMEC and 200 nM for MEF and HFFF. Tunicamycin (Merck, T7765) was used at 5 µg/ml.

### Transfection

A synthetic mutant *SEC61A1^D60G^* gene (Thermo Fisher Scientific) was generated and cloned into the pNLF1-C vector (Promega, N1361), replacing the nanoluciferase insert. The plasmid was digested with MluI (New England Biolabs, R0198S) and 2 µg transfected into HeLa cells with Fugene 6 (Promega, E2691) according to the manufacturer’s instructions. Cells were selected for one week in the presence of 10 ng/ml mycolactone epimers [80,81] (kind gift from Dr Nicolas Blanchard, CRNS France) and then for 1 week with 600 µg/ml hygromycin (Gibco, 10687010). The tandem tag vector pDEST-mCherry-eGFP-LC3B and control pDEST-mCherry-eGFP-C1 were a gift from Terje Johansen [31]. The plasmid named “HA-p62”, encoding hemagglutinin-tagged SQSTM1 was from Addgene (280277), deposited by Qing Zhong [82]. Plasmids (2 µg) were transfected into WT HeLa or PentaKO cells with Fugene 6. For tandem tag assays, WT cells were incubated at 37°C and 5% CO_2_ for 24 h, then trypsinized and plated onto sterile coverslips and incubated for a further 24 h. After treatment, cells were fixed with 4% paraformaldehyde (PFA; Merck, P6148) and mounted on Aqua-Polymount (Tebu-Bio Ltd, 18606–20) and imaged on a Nikon A1 confocal laser scanning microscope. Images were analyzed using ImageJ software (NIH). For the selection of stably transfected PentaKO cells expressing SQSTM1, resistant colonies that emerged after 2 weeks treatment with 400 µg/ml zeocin (Gibco, R25005) were picked, expanded and verified by western blotting.

### Small interfering RNA transfection

HFFF cells plated into 6-well plates were transfected with Dharmacon OnTarget Plus SMARTpool siRNAs (L-021117-00-0005 for *RB1CC1* and L-005049-00-0005 for *ULK1*) or OnTarget Plus Non-Targeting pool (D-001810-10-20) (see **Table S2** for details) according to manufacturer’s instructions using 10 µl siRNA and 4.5 µl DharmaFECT 1 Transfection Reagent (Horizon Discovery, T-2001) per well. Cells were incubated overnight then the medium was replaced. After a further 24 h the transfection was repeated. After overnight incubation, cells were trypsinized, plated onto coverslips and incubated overnight again before assaying.

### Immunofluorescence microscopy

Cells were grown on pre-sterilized glass coverslips. For LC3B visualization, cells were fixed and permeabilized in methanol (Merck, 34885-M) and blocked in 2% FBS, 1% goat serum (Merck, NS02L) in phosphate-buffered saline (PBS; Merck, 524650) for at least an hour. For all other markers, cells were fixed with 3.75% PFA in 200 mM HEPES (Merck, H0887), pH 7.5, permeabilized with 0.25% Nonidet P-40 Alternative in Netgel (150 mM NaCl [Thermo Fisher Scientific, S/3161/53], 5 mM ethylenediaminetetraacetic acid [Merck, E5134], 50 mM Tris-Cl [Thermo Fisher Scientific, BP152-1], pH 7.4, 0.05% Nonidet P-40 Alternative [Merck, 492016], 0.25% gelatin [Merck, G7401] and 0.02% sodium azide [Merck, S2002]). Antibodies used in this study are as follows: mouse anti-SQSTM1 (Abcam, 56416), rabbit anti-SQSTM1 (Enzo Biosciences, BML-PW9860), anti-LC3B (MBL International, PM036), anti-WIPI2 (Bio-Rad, MCA5780GA), anti-RB1CC1 (Bio-techne, NB100-77279), anti-ATG9A (Merck, ZRB1648), anti-ATG16L1 (Cell Signaling Technology, 8089), anti-NBR1 (Cell Signaling Technology, 9891), anti-CALCOCO2 (GeneTex, GTX115378), anti-TAX1BP1 (Proteintech 14,424-1-AP), anti-OPTN (Cayman Chemical, 100000), anti-rabbit Alexa Fluor 488 (Invitrogen, A11034; 1:500), anti-rabbit Alexa Fluor 594 (Invitrogen, R37117; 1:500), anti-mouse Alexa Fluor 488 (Invitrogen, A21202; 1:600). Nuclei were stained using a 0.1 µg/ml DAPI (Invitrogen, 62248) in PBS before mounting onto glass slides with VectaShield (Vector Laboratories, H-1000) or Aqua-Poly/mount. Slides were imaged on a Zeiss Axiovert S100 TV inverted fluorescence microscope or Nikon A1 confocal laser scanning microscope and NIS Elements software. Images were analyzed using ImageJ software. For quantification, puncta in each cell were counted using the ImageJ “Analyze Particles” function. This function measures number, area and intensity of fluorescent signals within a selected space. Where numbers of puncta are presented as averages in bar charts the number of puncta per field was divided by the number of nuclei. Where puncta per cell is represented by individual points, each point represents the number of puncta in an individual cell. For each experimental treatment puncta in each cell were counted for each experimental treatment in at least 3 fields.

### Cell lysis and immunoblotting

For analysis of proteins within detergent-soluble and -resistant fractions, cells were first lysed in RIPA buffer (Merck, 20–168) supplemented protease inhibitor cocktail (Merck, P9599), 1 mM sodium pyrophosphate (Merck, 71501), 5 mM sodium fluoride (Merck, S7920) and 1.75 mM β-glycerophosphate (Merck, G5422) for 5 min before centrifugation at 20,000 x *g* at 4°C. Supernatants (soluble) were removed and protein content was quantified by Pierce BCA assay (Thermo Fisher Scientific, 23227) before normalization to the lowest concentration. Pellets (insoluble) were dissolved in “gel sample buffer” (50 mM Tris, pH 6.8, 10% glycerol [Thermo Fisher Scientific, G/0650/17], 2.5% β-mercaptoethanol, 2% sodium dodecyl sulfate [Thermo Fisher Scientific, 1281–1680], 0.01% bromophenol blue [BDH Chemicals, 44305]) and sonicated for 30 s. Alternatively, whole cell lysates were prepared directly in gel sample buffer and sonicated for 30 s.

Lysates were resolved by sodium dodecyl sulfate-polyacrylamide gel electrophoresis alongside a broad range polypeptide marker (Spectra BR; Thermo Fisher Scientific, 26623) and transferred to PVDF membranes (Thermo Fisher Scientific, 11556345). Antibodies used in this study are as follows: anti-SQSTM1 (Abcam, Ab56416), anti-TUBB/β-tubulin (Bio-Techne Ltd, NB600-936SS), anti LC3B (Cell Signaling Technology, 4108), anti-EIF2AK3 (Cell Signaling Technology, 9606), anti-ubiquitin (FK2, Calbiochem, ST1200), anti-GAPDH (Ambion, AM4300), anti-rabbit-HRP (GE Healthcare, NA934V), anti-mouse-HRP (GE Healthcare, NA931V). Blots were developed using enhanced chemiluminescence with Immobilon™ western chemiluminescence HRP substrate (Thermo Fisher Scientific, 11556345) and imaged on a Fusion FX Imager (Vilber-Lourmat), which provides a warning if the areas of the image are saturated. Quantification was performed via ImageJ analysis of pixel density from non-saturated images and data are presented as normalized values to GAPDH controls.

### qRT-PCR

Total RNA was extracted using a QIAGEN RNeasy kit (74004) and real-time quantification of mRNA in triplicate samples was performed by TaqMan PCR on a QuantStudio7Flex real-time PCR system (Applied Biosystems) using the following program: 50°C for 2 min and DNA polymerase activation at 95°C for 10 min, followed by 40 cycles of at 95°C for 15 s and 60°C for 1 min. The following Applied Biosystems TaqMan probes were used in this study: *SQSTM1* (Mm_00448091_m1), *OSGIN1* (Mm_00660947_m1) and *GAPDH* (Mm_99999915_g1). Threshold cycle values were normalized to *GAPDH* internal controls and fold-change calculated from ΔΔCt values.

### Cell viability assays

Cell death induced by mycolactone was assessed using CellEvent™ Green Detection Reagent (Thermo Fisher Scientific, C10423) as previously described [16]. Briefly, cells were seeded in 96-well plates and treated as indicated, after which propidium iodide (Thermo Fisher Scientific, 11519206) and CellEvent were added at a final concentration of 0.3 μg/mL and 1% v:v, respectively. After a 30-min incubation at 37°C, 5% CO_2_, plates were imaged on a Zeiss Axiovert S100 TV inverted fluorescence microscope. For at least two technical replicates per condition, a total of 3–4 bright-field images were taken, as well as corresponding images under red (PI) and green (CE) fluorescence channels. The proportion of cells in late apoptosis (with PI^+^/CE^+^ nuclei) were expressed as a percentage of total cells enumerated from bright-field images (mean cell survival = $(1-\frac{CE{\;}_{\;}^{+}PI{\;}_{\;}^{+}nuclei}{total\;cells})\;X\;100$.

For viability assays (which measure both growth and death simultaneously), cells were seeded into 96-well plates at a density of 1000/well and treated with various concentrations of mycolactone for 96 h, then resazurin (Sigma Aldrich, R7017) added to a concentration of 0.01 mg/ml. After 4 h further incubation, emission at 620 nm at excitation 580 nm was measured using a BMG Fluostar Optima Microplate Reader. Data are expressed as a viability index, representing the relative signal compared to the negative control (DMSO-treated cells).

### Immunohistochemistry of human skin samples

Fixed and embedded 4 mm punch biopsies from Buruli ulcer patients were described previously [6]. In ulcerated BU lesions (6 patients) punch biopsies were taken 1 cm inside the outer margin of the induration surrounding the ulcer. For BU plaque lesions (2 patients) punch biopsies were collected from the non-ulcerated center of the lesion. Healthy control tissue was obtained at The Whitely Clinic or purchased from AMS Biotechnology (Europe) Ltd (500041028). After removal, all tissues were fixed in neutral formalin, embedded into paraffin (pfm Medical, 9000R2010) and sectioned (5 μm). Sections were deparaffinized, endogenous peroxidase quenched, epitopes unmasked (pH 6 citrate buffer; Sigma, C999) and blocked with horse serum (Vector Laboratories, S-2000-20). The tissue sections were then incubated with either anti-SQSTM1 antibody (Abcam, ab56416) or IgG2a isotype control (Santa Cruz Biotechnology, sc-3878) overnight at 4°C and biotin-conjugated secondary antibody (Vector Laboratories, BA2000). Staining was performed using VECTASTAIN Elite ABC kit (Vector Laboratories, PK-6100) and Vector NovaRED peroxidase substrate (Vector Laboratories, SK-4800). Counterstaining was performed with Shandon Harris Hematoxylin (Thermo Fisher Scientific, 6765001).

### Statistical analysis

All analyses were performed in GraphPad Prism v7: experiments assessing one independent variable across more than two groups were analyzed using a one-way analysis of variance (ANOVA), while those concerning two independent variables were analyzed by two-way ANOVA. Tukey’s post-hoc test was used to identify significant differences between groups and correct for multiple comparisons. Unless indicated with a line on the graph, statistical comparisons are to the DMSO-treated cells of that particular cell type. * = *p* < 0.05, ** = *p* < 0.01, *** = *p* < 0.001, ns = not significant.

### Ethics statement

Ethical approval for analyzing patient specimens was obtained from the Ethikkommission beider Basel, Basel, Switzerland and the provisional national ethical review board of the Ministry of Health Benin (N° IRB00006860) as well as from the Cameroon National Ethics Committee and the Ethics Committee of the Heidelberg University Hospital, Germany (ISRCTN72102977). Written informed consent from the patients or their guardians was obtained before specimens were collected for reconfirmation of BU as well as for detailed histopathological analysis and all patient data have been anonymized. Favorable ethical opinion for analyzing normal human skin was given by Faculty of Health and Medical Science Ethics Committee of the University of Surrey (1174-FHMS-16). The normal human skin samples were collected at The Whiteley Clinic or purchased from AMS Biotechnology (Europe) Ltd. Written informed consent was obtained from all donors. The research related to human tissues complies with the ethical processes of University of Surrey.

## Supplementary Material

Supplemental Material
