# Supplementary material for: Inhibition of the SEC61 translocon by mycolactone induces a protective autophagic response controlled by EIF2S1-dependent translation that does not require ULK1 activity
Source: Autophagy. 2021 Aug 23;18(4):841–59. doi: 10.1080/15548627.2021.1961067 (PMC9037441; doi:10.1080/15548627.2021.1961067)
Supplement: Supplemental Material [file KAUP_A_1961067_SM6401.zip › Hall et al editorial changes only supp data_R6_clean.doc]

**Hall et al Supplementary Figures and Figure Legends**

**Figure S1.** Mycolactone induces an increase in autophagic flux. Cells were exposed to mycolactone (Myco) at the minimum inhibitory concentration, alongside a solvent control (0.05% DMSO, equivalent dilution to the mycolactone) for the longest timepoint tested. All immunofluorescence images were obtained using a Nikon A1 confocal laser scanning microscope (scale bar: 10 µm). For all panels; ns = not significant; * = *p*<0.05; ** = *p*<0.01; *** = p<0.001; **** = p<0.0001. (**A**) HeLa cells were exposed to 31.25 ng/ml mycolactone for various times then fixed with methanol for LC3B staining. Values represent the number of puncta detected per cell for at least 150 cells. Data are representative of 3 independent experiments. (**B**) WT MEF were exposed to 31.25 ng/ml MYCL for 8 h in the presence or absence of 10 µM chloroquine (CQ) then fixed with methanol for LC3B staining. Values represent the number of puncta detected in each cell for at least 60 cells. Data are representative of 2 independent experiments. (**C**) Quantification of LC3B-II from western blot data for HFFF cells incubated with DMSO, 200 nM BAF for 4 h, 31.25 ng/ml Myco for 24 h, or 31.25 ng/ml Myco for 24 h with the addition of BAF for the final 4 h. Values represent the means of three independent experiments and are normalized to TUBB (left hand panel) and presented as a percentage of control, or the ratio of LC3B-II to LC3B-I (right hand panel). (**D**) Deconvolution of images shown in **Fig. 1D**. (**E**) Expression of untagged mCherry-GFP in HeLa cells. Cells transiently transfected with pDEST-mCherry-eGFP were exposed to 31.25 ng/ml Myco for 4 h before the addition of 100 nM (BAF) and incubation for a further 4 h before fixation with 4% PFA in PBS. (**F**) HeLa cells, HDMEC and HFFF cells exposed to different concentrations of bafilomycin A_1_ (BAF) for 4 h were lysed directly in gel sample buffer prior to immunoblotting. The migration relative to known molecular mass markers is shown. (**G**) WT MEFs were exposed to 31.25 ng/ml mycolactone for various times, then fixed for WIPI2 staining. Values represent the mean of 3 independent experiments ± SEM. (**H**) WT MEF and HeLa cells were exposed to 31.25 ng/ml mycolactone for 8 h then fixed and stained for WIPI2. Data are representative of 3 independent experiments ± SD. (**I**) Colocalization of WIPI2 and RB1CC1 puncta in WT MEF exposed to mycolactone for 8 h. Cells were fixed and co-stained for WIPI2 and RB1CC1 (scale bars: main image: 10 µm, inset: 2 μm). (**J**) HFFF were exposed to DMSO or 31.25 ng/ml mycolactone for 8 h, or PP242 then fixed for RB1CC1 or ATG16L1 staining. Values represent the puncta detected in each cell for 38-160 cells. Data are representative of duplicate experiments.

**Figure S2.** Accumulation of SQSTM1 and ubiquitin in mycolactone exposed cells. Cells were exposed to 31.25 ng/ml mycolactone (Myco), alongside a solvent control (0.05% DMSO, equivalent dilution to the mycolactone) for the longest timepoint tested. For all immunoblots, the migration relative to known molecular mass markers is shown. All immunofluorescence images were obtained using a Nikon A1 confocal laser scanning microscope. (**A**) HeLa cells were exposed to mycolactone for the times indicated with or without 100 nM bafilomycin A_1_ (BAF) for 4 h then fixed with PFA for SQSTM1 staining with a mouse anti- SQSTM1 antibody (scale bar: 10 µm). Values represent the number of puncta detected per cell for 60-160 cells. Data are representative of 2 independent experiments

(**B**) Deconvolution of images shown in **Fig. 2C** (scale bar: 10 µm). (**C**) RAW264.7 or (**D**) WT MEF cells were left untreated, or exposed to mycolactone, for different times (as indicated) or 50 µM chloroquine (CQ) for 12 h. The 48-h timepoint was performed without and with 50 µM CQ (for the final 12 h). Cells were harvested in RIPA lysis buffer, then soluble and insoluble fractions were prepared as described in Methods prior to immunoblotting. Data are representative of 3 independent experiments. (**E**) HFFF were exposed mycolactone for 8 h then fixed with PFA for staining with anti-pan-ubiquitin (green) and rabbit anti-SQSTM1 (red) antibodies (scale bar: 10 μm).

**Figure S3.** Generation of mycolactone-resistant HeLa cells by expression of mutant *SEC61A1* gene, and lack of role for ATG9A in mycolactone response. (**A-C**) HeLa cells stably overexpressing the Flag-tagged SEC61A1 mutant, SEC61A1^D60G^ were generated by selection with 10 ng/ml mycolactone (Myco) epimers for one week, followed by hygromycin for one week. Cells are compared to untransfected cells (WT). (**A**) Untreated cells were lysed directly in gel sample buffer prior to immunoblotting. Migration relative to known molecular mass markers is shown. (**B**) Untreated cells stained for FLAG tag (scale bar: 10 μm). (**C**) Cell viability after 4 d exposure to mycolactone was assessed by resazurin assay. Values represent the mean of triplicate assays ± SD and are representative of three independent experiments assays. The IC_50_ for wild type cells was 2.95 ng/ml. (**D**) HFFF cells exposed to 31.25 ng/ml mycolactone for various times were lysed directly in gel sample buffer prior to immunoblotting. The migration relative to known molecular mass markers is shown. Data is representative of 2 independent experiments. (**E**) HFFF were exposed to 31.25 ng/ml mycolactone for 8 h or 1 μM PP242 for 1 h then fixed for ATG9A staining. Data is representative of 2 independent experiments (scale bar: 10 μm).

**Figure S4.** Role of SQSTM1 and other autophagy receptors in survival of cells exposed to mycolactone. Cells were exposed to 31.25 ng/ml mycolactone (Myco), alongside a solvent control (0.05% DMSO, equivalent dilution to the mycolactone). All immunofluorescence images were obtained using a Nikon A1 confocal laser scanning microscope (scale bar: 10 µm). For immunoblots, the migration relative to known molecular mass markers is shown. For all panels, ns = not significant; * = *p*<0.05; ** = *p*<0.01; *** = p<0.001; **** = p<0.0001. (**A**) Matched *Sqstm1* WT and *sqstm1^-/-^* MEFs cells were exposed to mycolactone for 8 h before fixing with PFA for immunofluorescent staining with mouse anti-SQSTM1, anti-TAX1BP, anti-NBR1 or anti-OPTN (optineurin) antibodies. Data are representative of duplicate experiments (scale bar: 10 µm). (**B**) Matched *Sqstm1* WT and *sqstm1^-/-^* MEFs were exposed to mycolactone for the indicated times. Cell viability as measured by CellEvent assay. Values represent the mean of 3 independent experiments ± SEM. (**C**) WT and PentaKO (5KO) HeLa cells were exposed to mycolactone for the indicated times. Cell viability as measured by CellEvent assay. Values represent the mean of 3 independent experiments ± SEM. (**D**) WT and PentaKO (5KO) HeLa cells were exposed to mycolactone for 16 h or 10 μM MG132 for 8 h then fixed with PFA for staining for total ubiquitin with FK2 (scale bar: 10 µm). (**E**) WT HeLa, PentaKO (5KO) HeLa cells, and PentaKO cells with reconstituted SQSTM1 expression (5KO/*SQSTM1*) were lysed directly in gel sample buffer prior to immunoblotting. The migration relative to known molecular mass markers is shown.

**Figure S5.** Mycolactone stimulates an ULK-independent, RB1CC1-dependent pathway of autophagosome biogenesis. Cells were exposed to 31.25 ng/ml mycolactone (Myco), alongside a solvent control (0.05% DMSO, equivalent dilution to the mycolactone). PP242 (1 µM) for 1 h was used as a control. For all panels, ns = not significant; * = *p*<0.05; ** = *p*<0.01; *** = p<0.001; **** = p<0.0001. (**A** and **B**) Matched *Rb1cc1* WT and *rb1cc1^-/-^* MEF were exposed to mycolactone for 8 h. (**A**) Cells were fixed with PFA and stained for RB1CC1. (**B**) Cells were fixed with PFA for SQSTM1 staining with mouse anti-SQSTM1 antibody, and the area of the SQSTM1 puncta was assessed using ImageJ software. (**C** and **E**) Matched *Rb1cc1* WT and *rb1cc1^-/-^* MEFs (**C**), or matched *Ulk1-Ulk2* WT and *ulk1^-/-^ ulk2^-/-^* MEFs (**E**) were exposed to mycolactone for 24 h. Cell viability as measured by CellEvent assay. Values represent the mean of 3 independent experiments ± SEM. (**D**) Quantification for **Fig. 5C**. WT MEFs were lysed directly in gel sample buffer prior to immunoblotting. Band intensity was determined using ImageJ software. Values are the ratio of phosphorylated to total ULK1 proteins, presented as percentage of control and represent the mean of triplicate experiments ± SEM.

**Figure S6.** Mycolactone-induced autophagy markers are dependent on the integrated stress response. Cells were exposed to 31.25 ng/ml mycolactone (Myco), alongside a solvent control (0.05% DMSO, equivalent dilution to the mycolactone) for the longest timepoint tested. When needed, 5 µg/ml tunicamycin (Tuni) for 6 h, or 1 µM PP242 for 1 h were used as controls. Where used, bafilomycin A_1_ (BAF) was added at saturating concentration (100 nM) for 4 h. For all immunoblots, the migration relative to known molecular mass markers is shown. For all panels, ns = not significant; * = *p*<0.05; ** = *p*<0.01; *** = p<0.001; **** = p<0.0001. (**A**) Matched parental *Eif2ak3* WT and *eif2ak3^-/-^* MEFs exposed to DMSO or mycolactone for 24 h with or without BAF were lysed directly in gel sample buffer prior to immunoblotting. (**B**) Matched parental *Eif2ak3* WT and *eif2ak3^-/-^* MEFs were exposed to mycolactone as indicated or 100 μM MMF for 24 h. Total RNA was extracted from cells and subjected to qRT-PCR; data were normalized to *Gapdh* mRNA. Values represent the mean for three independent experiments ± SEM. (**C**) WT MEF were exposed to mycolactone for 6 h in the presence or absence of 200 nM ISRIB, then lysed with gel sample buffer prior to immunoblotting; the migration relative to known molecular mass markers is shown. (**D**) Matched parental *Eif2s1* WT and *eif2s1^S51A^* MEF were exposed to mycolactone for 8 h. Cells were fixed with 3.75% PFA in 200 mM HEPES, pH 7.5 for WIPI2 and RB1CC1 staining, or with methanol for LC3B staining. Data represent the mean of at least 3 independent experiments ± SEM. (**E**) Matched parental *Eif2s1* WT and *eif2s1^S51A^* MEF were exposed to mycolactone for 6 h then lysed directly in gel sample buffer prior to immunoblotting. (**F**) Matched parental *Eif2s1* WT and *eif2s1^S51A^* MEF were exposed to mycolactone for 24 h. Cell viability as measured by CellEvent assay. Values represent the mean of 3 independent experiments ± SEM.

**Table S1.** Characteristics of Buruli ulcer patients and SQSTM1 staining pattern.

| Patient | WHO Category | Lesion Type | SQSTM1-positive staining seen in | |
| --- | --- | --- | --- | --- |
|  |  |  | Inflammatory cells | Sub-endothelial cells |
| 1 | 1 | U | + | - |
| 2 | 2 | U | + | - |
| 3 | 2 | U | + | - |
| 4 | 1 | U | + | - |
| 5 | 3 | U | + | - |
| 6 | 3 | P | + | + (imaged) |
| 7 | 2 | U | + (imaged) | - |
| 8 | 2 | P | + | + (imaged) |

U; Ulcer

P; Plaque

+; positive staining

-; no staining seen above background

**Table S2.** siRNA oligonucleotides.

| **TARGET** | **siRNA** |
| --- | --- |
| **NT** | UGGUUUACAUGUCGACUAA  UGGUUUACAUGUUGUGUGA  UGGUUUACAUGUUUUCUGA  UGGUUUACAUGUUUUCCUA |
| ***ULK1*** | CAGCAUCACUGCCGAGAGG  CCACGCAGGUGCAGAACUA  GCACAGAGACCGUGGGCAA  UCACUGACCUGCUCCUUAA |
| ***RB1CC1*** | GGAGUGGGCUGGUGCUUUA  AAACUACGAUUGACACUAA  GCAAAGAAAUUAGGGAAUC  UAAACUUGACGGACUAAUA |
